# Chronic wounds treated with cold atmospheric plasmajet versus best practice wound dressings: a multicenter, randomized, non-inferiority trial

**DOI:** 10.1038/s41598-022-07333-x

**Published:** 2022-03-07

**Authors:** R. Strohal, S. Dietrich, M. Mittlböck, G. Hämmerle

**Affiliations:** 1 Department of Dermatology, Federal Academic Teaching Hospital Feldkirch, Carinagasse 45-47, 6800 Feldkirch, Austria; 2 Central Wound Center at the Department of Dermatology, Federal Academic Teaching Hospital Feldkirch, Feldkirch, Austria; 3 Center for Medical Statistics, Informatics, and Intelligent Systems, Section for Clinical Biometrics, Medical University of Vienna, Vienna, Austria; 4 Central Ambulance of Wound Care, Department of Nursing, Academic Teaching Hospital Bregenz, Bregenz, Austria

**Keywords:** Trauma, Therapeutics

## Abstract

The use of phase-adapted wound dressings represents best practice (BP) in chronic wound treatment. However, efficacy is often limited and associated care requirements are high. Cold atmospheric plasmajet (CAP-jet) is a promising new therapeutic tool for these wounds. In the present multicenter, randomized, open-label, prospective, clinical trial, non-inferiority of the CAP-jet versus BP was assessed in 78 patients with infected or non-infected chronic wounds of different etiology. Primary outcome measure was the sum of granulation tissue, furthermore wound area reduction, healing rate, time to complete healing, changes in wound pH value, infection score, exudate level and local tolerability were assessed. In CAP-jet treated wounds compared to control, the sum of granulation tissue was significantly higher (p < 0.0001) and wound area reduced significantly faster (p < 0.001). Furthermore, wound pH value decreased significantly faster (p = 0.0123) and local infection was overcome more rapidly by CAP-jet therapy. In 58.97% CAP-jet- vs. 5.13% BP-treated patients, complete healing of chronic ulcers was documented after 6 weeks. Treatment with CAP-jet appeared not only non-inferior, but even superior to BP in all wound entities analyzed with a favorable tolerability profile. Thus, treatment with the CAP-jet provides beneficial effects in chronic wound treatment regarding promotion of the wound healing process.

## Introduction

Up to 5% of the general population in developed countries are affected by chronic wounds such as diabetic foot ulcers (DFU), venous leg ulcers (VLU), peripheral arterial occlusive disease (PAOD)-associated wounds and pressure ulcers (PU)^1–3^. Older people are particularly at risk for the development of such diseases, and due to the demographic change in the population a rising incidence can be assumed in future^4^.

In general, chronic wounds are linked to a high morbidity and impact on patients’ quality-of-life^5–7^. The management of these wounds is often a major challenge in daily clinical practice. Wound healing processes are complex and depending on the healing phase, hence, different dressings with adapted properties are used within the framework of the current standard of care (SOC)^1,8^. In consequence, wound phase-adapted treatment requires an ongoing reorientation within the range of products, and individual treatment methods suitable per healing phase often based on the practitioners’ experience and the best practice (BP) used. Additionally, local wound infections can result in a further healing delay and increase the risk of critical systemic infections^9^. Management of such infections requires a broad range of different wound dressings.

The reasons for delayed or stagnant wound healing are varying and include different factors of the wound microenvironment. However, present therapeutic options to overcome these problems are limited. Cold atmospheric plasma (CAP) is a new therapeutic approach used for various medical purposes^10–12^. CAP is composed of free electrons and ions, electromagnetic fields, a certain amount of visible and ultraviolet light and infrared radiation as well as reactive oxygen species (ROS), nitric oxide (NO), hydrogen peroxide (H_2_O_2_), hydroxyl radicals (HO·) and atomic oxygen. These molecules are responsible for the broad biological efficiency of CAP on regenerative cellular processes^12^. Amongst others, disinfecting as well as a pH-modulating effect have been described^13^. Both aspects represent very important issues in wound healing. Chronic wounds are susceptible for bacterial and fungi infections, which in turn can promote delayed wound healing and enhance the risk for the development of dangerous systemic infections^14,15^ The physiological acidic pH value of the skin is essential for its complete functionality and resistance to noxae. In the process of wound healing, changes in the pH value are prerequisite to control enzyme activity and induce wound healing. However, chronic wounds are often characterized by an alkaline milieu, impairing not only the physiological wound healing processes but also promoting colonization with human-pathogenic bacteria^15^.

Due to its physical properties, CAP can directly be applied to cells or tissues^11,13,16^.

Various technical approaches exist to generate plasma devices for medical use. The on the market available cold atmospheric plasmajet kINPen^®^ MED (CAP-jet) is based on the jet principle and intended for indirect plasma treatment. In contrast to other devices available on the market, it operates with argon gas, guaranteeing a controlled plasma composition with a constant quality. It is characterized by a fine plasmajet, which can be precisely guided into the smallest gaps and pores of the skin^12,17^. The therapeutic effects of argon-based cold plasma are induced by reactive oxygen and nitrogen species, an irradiation in the UV light range, and a topical short-term increase in temperature.

Other CAP concepts on the market are based on Dielectric-barrier discharge (DBD), in which the plasma is generated from the ambient air. DBD devices compose a planar but inflexible and practically unadaptable plasma layer. These devices are used to treat larger skin wounds if they are not as fissured, jagged and with undercuts as ulcerative, traumatic and surgical wounds are in general.

A number of clinical trials using different devices have been performed so far. However, due to the different product concepts and physical mechanisms of action, study results for individual application can only be transferred to a limited extent^18,19^. In a phase I study, the antibacterial efficacy of a DBD-based CAP device has been confirmed^20^. A multiple treatment proved harmless, and compared to the control group a significant reduction of bacterial colonization could be detected. Furthermore, treatment promoted cell proliferation and neovascularization^21,22^. In two phase II randomized controlled trials (RCT), microwave-based application proved safe with a high antimicrobial potential in patients with various chronic wounds, respectively^23,24^.

The first placebo-controlled, patient-blinded RCT to investigate the effect of the argon-based plasmajet on wound area reduction as well as infection status and microbial load of chronic wounds was performed by Stratmann et al.^25^. In this study, 65 DFU from 45 patients treated either with SOC + CAP-jet or SOC + placebo were analyzed^25^. As a result, a significant increase in wound healing (p = 0.03) and faster wound area reduction from baseline (p = 0.009) in favor of CAP-jet compared to placebo was reported^25^. Furthermore, a reduction of microbial load was detected in both groups. CAP-jet treatment proved well tolerated for patients, in addition, no treatment-associated adverse events occurred until the end of the study. In this RCT, it was demonstrated for the first time that treatment with the CAP-jet can stimulate the healing process in chronic wounds and thus contributes to faster wound closure. These results support previous findings from other studies performed with the CAP-jet^26,27^. In a small study including 34 patients with chronic leg ulcers the antibacterial effect of CAP-jet treatment was demonstrated^26^. Furthermore, in a case series the results for the use of CAP-jet in common wound situations in daily clinical practice were presented for wounds of different origin^27^. In the case of infected wounds, CAP-jet therapy led to a reduction of both bacterial load and fibrin coatings, in inflammatory wounds a reduction or even elimination of inflammation have been observed. Furthermore, granulation and epithelialization were stimulated.

Proof of concept for the efficacy and safety of CAP application on chronic wounds with a jet was also provided by Mirpour et al.^28^. For a helium-based system, the authors demonstrated that already after 3 weeks the number of wounds reaching a fraction wound area of ≤ 0.5 was significantly greater after treatment with SOC + CAP compared to SOC alone (p = 0.006)^28^. Furthermore, CAP was favorable in reduction of bacterial load in infected wounds (p < 0.01).

Based on the promising CAP-jet data existing so far, the present multicenter, randomized, open-label, prospective, non-inferiority clinical trial aimed to investigate the healing efficacy of the cold atmospheric plasmajet kINPen^®^ MED in wounds with different etiology compared to BP wound phase-adapted treatment in the controlled setting of a RCT. Another aspect was to examine the antimicrobial effect of CAP-jet treatment in a larger patient setting. The trial was designed to evaluate patient-relevant efficacy and tolerability indicators in the treatment of infected and non-infected wounds with the primary hypothesis that treatment with the CAP-jet is non-inferior to BP in terms of granulation tissue formation in wound healing at the end of study.

## Results

### Patient enrollment and withdrawals

**Figure 1:**
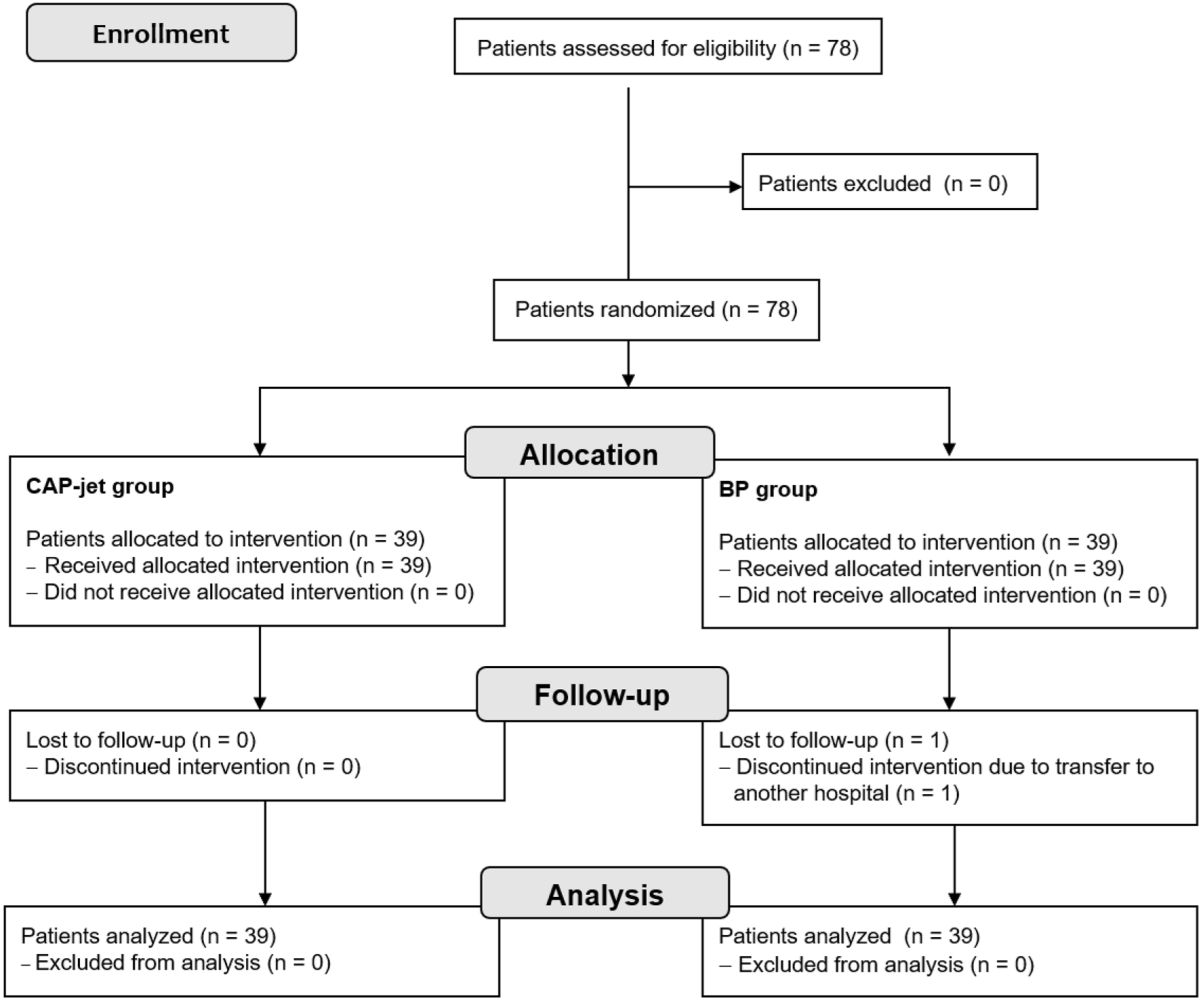
Study flow chart.

After testing the eligibility for participation in the study, all 78 patients were included and randomly allocated to the intervention group receiving treatment with either a CAP-jet or BP modern wound dressings (Fig. 1). Seventy-seven patients (98.72%) completed the 6-week treatment period, 39 (100%) patients in the CAP-jet group and 38 (97.44%) patients in the BP group, respectively.

### Demographics of patients and characteristics of target ulcers

Fifty-six (71.79%) patients were males and 22 (28.21%) females, median (min; max) age was 69.18 (37.08; 93.91) years (Table 1).

**Table 1:** Patient demographics and disease characteristics at baseline visit.

	CAP-jet group n = 39 (100%)	BP group n = 39 (100%)	Total N = 78 (100%)
**Demographic characteristics**			
Age in years, median (min; max)	72.87 (42.58; 93.91)	67.99 (37.08; 92.30)	69.18 (37.08; 93.91)
Age in years, mean (SD)	70.34 (14.84)	67.11 (14.56)	68.73 (14.69)
Males, n	31 (79.49)	25 (64.1)	56 (71.79)
Females, n	8 (20.51)	14 (35.90)	22 (28.21)
**Wound etiology, n (%)**			
VLU	22 (56.41)	21 (53.85)	43 (55.13)
PAOD	5 (12.82)	2 (5.13)	7 (8.97)
Mixed leg ulcers	5 (12.82)	12 (30.77)	17 (21.79)
DFU	7 (17.95)	3 (7.69)	10 (12.82)
PU	–	1 (2.56)	1 (1.28)
**Wound characteristics**			
Ulcer onset, in months, median (min; max)	3.50 (1.00; 11.00)	3.00 (0.00; 9.00)	3.00 (0.00; 11.00)
Ulcer onset, in months, mean (SD)	4.13 (2.35)	3.69 (2.09)	3.91 (2.22)
Wound area in cm^2^, median (min; max)	3.52 (0.26; 24.26)	3.84 (0.48; 45.15)	3.68 (0.26; 45.15)
Infection, n (%)	13 (33.33)	18 (46.15)	31 (39.74)
No infection, n (%)	26 (66.66)	21 (53.85)	47 (60.26)

*BP* best practice, *CAP* cold atmospheric plasma, *DFU* diabetic foot ulcer, *max* maximum, *min* minimum, *N* total number of patients, *n* group size (number of patients with event), *PAOD* peripheral artery occlusive disease, *PU* pressure ulcer, *SD* standard deviation, *VLU* venous leg ulcer.

Wound etiology comprised 43 (55.13%) VLU, 7 (8.97%) PAOD-derived ulcers, 17 (21.79%) ulcers of mixed etiology, 10 (12.82%) DFU and 1 (1.28%) PU. Infection was present in 31 (39.74%) wounds, 47 (60.26%) were free of infection. Ulcer onset lasted in median (min; max) 3 (0.00; 11.00) months. Median (min; max) wound area was 3.52 (0.26; 24.26) cm^2^ in the CAP-jet group and 3.84 (0.48; 45.15) cm^2^ in the BP group.

Median (min; max) wound pH value at baseline amounted to 9.93 (7.36; 11.97) and 9.98 (9.13; 11.49) in the CAP-jet group and BP group, respectively.

### Sum of granulation tissue at end of study

**Figure 2:**
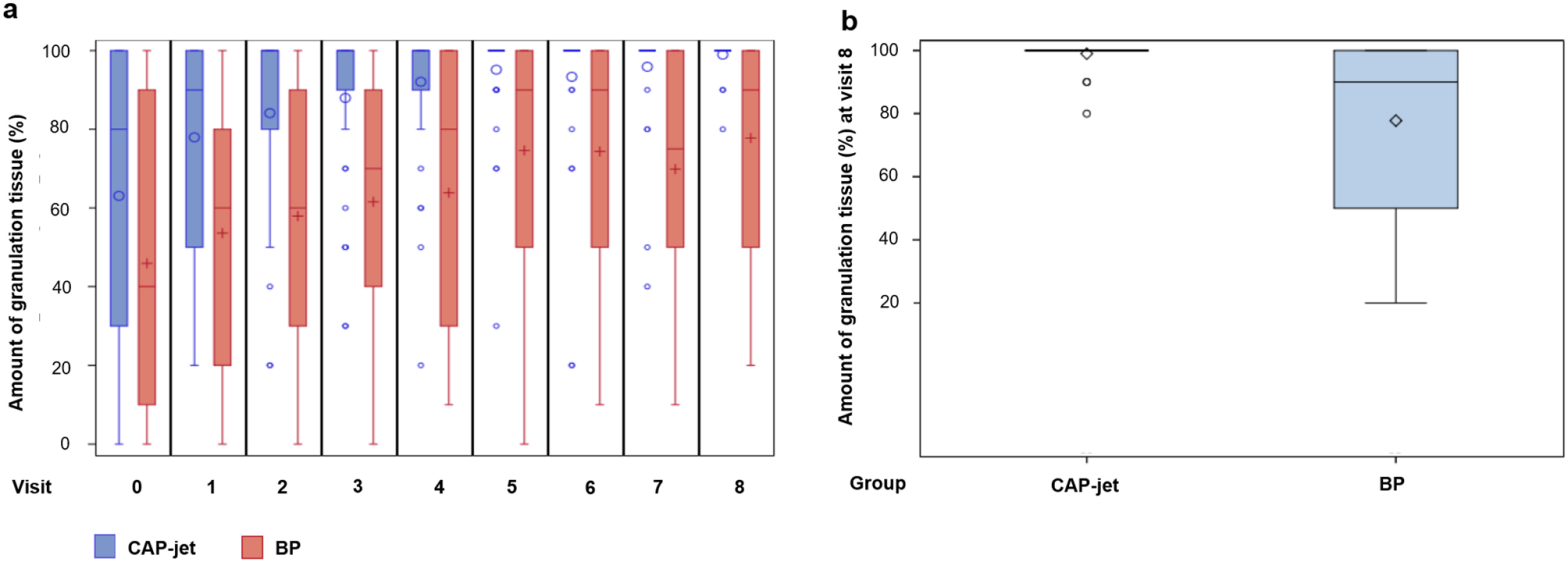
Percentage of the sum of granulation tissue; (**a**) dynamics (%) of granulation tissue, (**b**) amount (%) of granulation tissue at end of study (v 8, d 42). In case of 100% granulation tissue, equaling complete healing, 100% has been imputed for all following visits until end of study (v 8, d 42). All data available of one patient dropped-out have been included.

In both treatment groups, the percentage (%) of granulation tissue increased over time (Fig. 2a, Supplementary Table S1). For the CAP-jet group, the mean ± standard error (SE) proportion increased from 63.08 ± 5.68 at d 0 to 98.97 ± 0.61% at d 42 (v 8). In the BP group, an increase (mean ± SE) from 45.90 ± 5.83 to 77.76 ± 4.49% was detected, respectively. In contrast, wounds in the CAP-jet group reached mean ± SE 95.13% ± 2.11% granulation tissue, corresponding to almost complete healing, already at d 21 ± 2 (v 5) vs. BP with mean ± SE 74.62% ± 4.59%, respectively.

At the end of study (v 8), non-inferiority was proven for CAP-jet over BP treatment with a mean difference of 21.21 percentage points (95% confidence interval [13.57; ∞]; p < 0.0001; Fig. 2b) using a one-sided unpaired t-test with non-inferiority margin of − 15 percentage points. Furthermore, using a t-test with a two-sided 95% confidence interval (CI) in analogy to the non-inferiority testing, CAP-jet treatment reached even superiority over BP with a difference of means between groups of 21.21% (95% CI [12.04, 30.38]; p = 0.001).

However, comparing the baseline values of CAP-jet and BP revealed a statistically significant difference in favor of BP with mean ± SE 63.08 ± 5.68% vs. 45.90 ± 5.83% (p = 0.0394). Adjustment for baseline values using a mixed linear model with repeated measurements (visits) per patient assuming a first-order autoregressive variance–covariance matrix confirms the statistically significant difference between the groups in favor of CAP-jet (p < 0.0001). Thus, increase over time was statistically significant faster with CAP-jet treatment compared to BP.

### Secondary endpoints

#### Wound area at end of study

**Figure 3:**
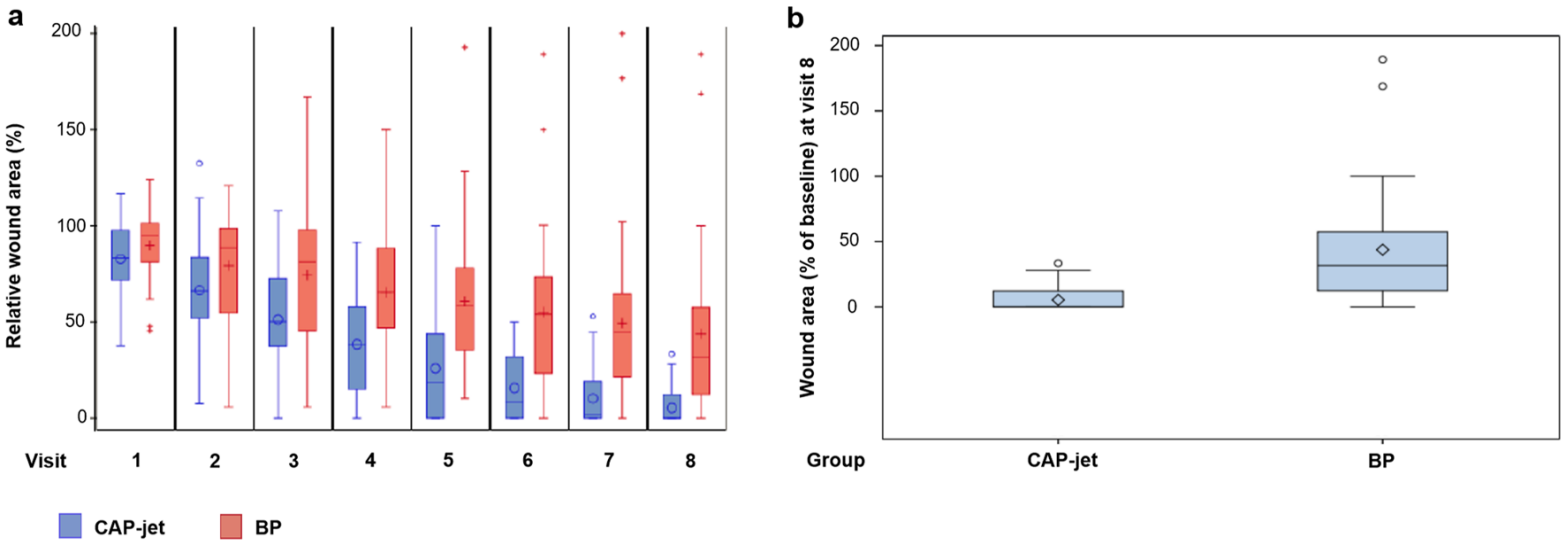
(**a**) Relative wound area in % from baseline; (**b**) distribution of the relative change in wound area (%) at end of study (v 8, d 42). A value of 0% (0 cm^2^) has been considered as healing and was imputed when healing was diagnosed as well as at all following visits until v 8 (d 42). All data available of one patient dropped-out have been included. No information after drop-out was available.

At baseline, wound area was comparable between treatment groups (median [min; max] 3.52 cm^2^ [0.26; 24.26] for CAP-jet vs. 3.84 cm^2^ [0.48; 45.15] for BP, respectively). The relative wound area from v 0 to v 8 decreased significantly in both groups (p < 0.0001 each). However, the relative wound area under CAP-jet therapy decreased statistically significant faster over time than under BP treatment (p < 0.001; Fig. 3a, Supplementary Table S1).

At end of study (v 8), wound area percentage from baseline has improved for CAP-jet treatment with mean ± SE of 5.32 ± 1.39 vs. 43.72 ± 6.98 in the BP group (Supplementary Table S1). The unpaired t-test revealed that CAP-jet treatment is non-inferior to BP treatment with a 15 percentage points non-inferiority margin. In fact, the mean difference from v 0 to v 8 was 38.40% lower in Cap-jet than in the BP group (95% CI [− ∞; − 26.4], p < 0.0001; Fig. 3b).

Furthermore, a two-sided unpaired t-test for superiority showed significantly better results for wound area dynamic from baseline to d 42 (v 8) for the CAP-jet compared to BP (mean difference 38.40%; two-sided 95% CI [− 52.80, − 24.01]; p < 0.0001).

#### Infection control

At baseline (v 0), 13 (33.33%) patients in the CAP-jet and 18 (46.15%) patients in the BP group had infected wounds (Table 1). In the CAP-jet group, all 13 infected wounds registered at v 0 had healed by the end of the study (v 8); one newly infected wound emerged at v 8 (Supplementary Table S1). In the BP group, 14 of initially 18 (77.78%) infections had resolved by v 8, 4 (22.22%) infections persisted.

**Figure 4:**
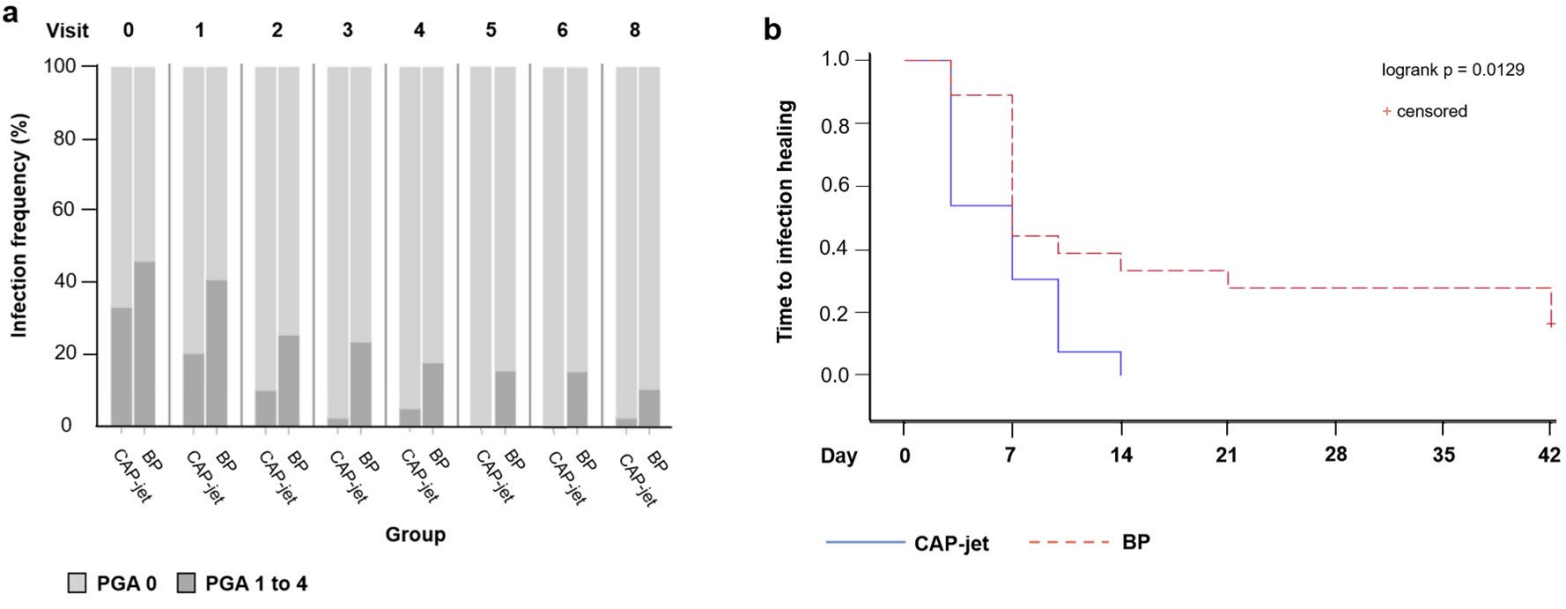
(**a**) Frequency of infection; (**b**) time to infection healing. (**a**) The newly emerged infection in CAP-jet group at v 8 was not imputed; in the BP group, one value at v 3 of one patient is missing, furthermore, the values of the patient who was transferred to another hospital after v 6 are missing in v 7 and v 8. Healed patients are considered as no infection.

In both treatment arms, wound infection from v 0 to v 8 decreased statistically significant in the course of therapy with p = 0.0013 for CAP-jet and p = 0.0002 for BP, respectively (Fig. 4a). After adjusting for the higher baseline infection count of the BP arm and inclusive the new infection at v 8 in the CAP-jet group, the difference between numbers of infections at v 8 in the CAP-jet arm vs. the BP arm differed not significantly (p < 0.2598) in favor of the CAP-jet. However, since the new infection that occurred in the CAP-jet group at v 8 no longer received cold plasma treatment due to the end of the study, it had to be excluded from the study calculations, revealing a significantly (p < 0.0129) shorter time to resolving the infections in patients receiving CAP-jet treatment than in patients receiving BP (Fig. 4b). The PGA infection score stratified by visit correlated statistically significant (p = 0.0334) with the exudate level.

#### Reduction of wound pH value

**Figure 5:**
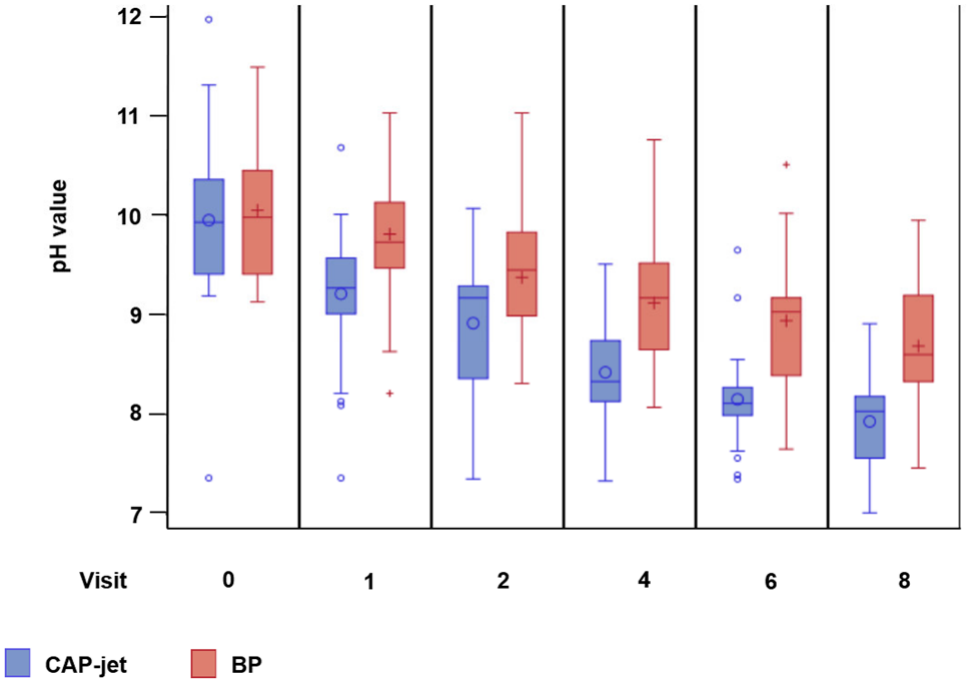
Dynamics of pH values from baseline to end of study at visit 8 (d 42). Available data of patients dropped-out have been included, no values have been imputed at that visit when healing was diagnosed and all following visits until d 42.

Comparison of mean ± SE wound pH value at baseline revealed comparable results in both treatment groups (CAP-jet 9.95 ± 0.12, BP 10.05 ± 0.11). Values decreased statistically significant over time and reached mean ± SE values of 7.93 ± 0.12 (p < 0.0001, CAP-jet) and 8.69 ± 0.10 (p < 0.0001, BP) at end of the study (d 42), respectively (Supplementary Table S1, Fig. 5). However, the wound pH value under CAP-jet therapy decreases significantly faster than under BP (p = 0.0123) even though data of healed wounds, which were more common with CAP-jet treatment, were not imputed in the evaluation.

#### Healing rate and time to complete healing

With CAP-jet treatment, wounds of 23 (58.97%) patients had healed at the end of study, while only 2 (5.13%) wounds of patients receiving BP treatment were healed (Supplementary Table S1). The time to complete healing was significantly shorter with CAP-jet than with BP therapy (p < 0.0001).

#### Exudate management

**Figure 6:**
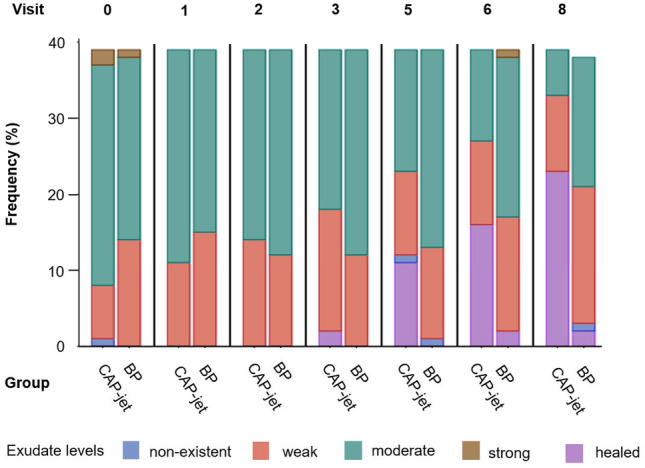
Exudate levels over time. Values of the patient in the BP group who was transferred to another hospital after v 6 are missing in v 8.

At baseline (v 0), 2.56% of wounds in the CAP-jet group displayed no exudate, in 17.95% the level was estimated low, in 74.36% moderate and in 5.13% high exudate levels were detected (Fig. 6). For the BP group, exudate levels were low (35.90% of wounds) to moderate (61.54% of wounds) in most patients. In 2.56% of wounds, the exudate levels were high. Until study end (d 42), exudate management improved in both groups. Of the remaining 16 patients in the CAP-jet group with non-healed wounds, in 62.50% and 37.50% the exudate levels were low or moderate, respectively. In the BP group, 2.78% of the 36 patients still suffering from their wound displayed no exudate, while in 50.00% and 47.22% the exudate levels were low or moderate, respectively.

#### Treatment-associated sensation during CAP treatment

During the study period, a total of 278 surveys regarding the patients` sensation during the administration with the cold plasmajet on the wound were evaluated. In 51.90% of all observations, patients even described the CAP-jet treatment as comfortable (Supplementary Table S1).

#### Safety assessment: local tolerability and adverse events

To determine local tolerability, presence or absence of erythema, maceration, blisters and congestion of exudate were assessed. While in the CAP-jet group none of the disorders mentioned occurred (n = 0), in the BP group three patients suffered from erythema; furthermore, wounds of three patients showed signs of maceration and in further two cases other disorders were detected (Supplementary Table S1). In both groups, no AE as well as SAE occurred during the study period.

## Discussion

Chronic wounds and the development of critical wound infections represent growing challenges in daily clinical routine. To our knowledge, the existing studies on the effect of CAP treatment have only been performed with CAP in combination with additional SOC or BP therapy. The present RCT is the only one that investigates in the effect of CAP treatment without additional measures on the healing of infected as well as non-infected chronic wounds of different origin. For the first time, solely the effect of a CAP device is compared to clinical daily best practice treatment without the undermining effect of complementing therapeutic measures, allowing the evaluation of efficacy and safety of the CAP-jet as independent treatment strategy without application of additional SOC or BP therapy.

The results presented here demonstrate the good efficacy of the chosen BP consisting of wound phase-adapted dressings and antimicrobial therapy. Relative wound area as well as wound pH values decreased statistically significant from baseline to the end of study (p < 0.0001 each), the number of wound infections was significantly lower at the end of study compared to baseline as well (p = 0.0002). These results indicate that the dressings used in BP were selected adequately and according to the individual wound requirements.

However, by using the CAP-jet even better results could be achieved. The treatment was not only non-inferior to the BP of modern, wound phase-adapted dressings but also clearly exceeded it with significant superiority. Increase of granulation tissue over time was statistically significant faster (p < 0.001). Additionally, relative wound area with CAP-jet therapy decreased statistically significant faster over time than with BP treatment, and at the end of the study the mean difference from v 0 to v 8 was favorable for the CAP-jet (both p < 0.0001). A statistically significant improvement in comparison to BP was also achieved regarding the time to complete healing (p < 0.0001). As with BP, under CAP-jet treatment the number of infected wounds decreased significantly over time (p = 0.0013). However, an additional use of antiseptics was not necessary while in the control group, despite the use of antiseptics, not all infections could be eliminated within study duration. Importantly, time until resolving the infections proofed statistically significant shorter under therapy with CAP-jet treatment (p < 0.0129). It has to be noted that the newly emerged infection at v 8 in the CAP-jet group was not imputed into the analysis of time to resolve infection, as the study ended at the time point (v 8) when the infection occurred and CAP-jet treatment could not be applied for that specific wound. In addition to the data for the good control of wound-associated infections, therapy with the CAP-jet also resulted in a favorable exudate management (no maceration in the CAP-jet group vs. 3 patients in the BP group) and a significantly faster wound pH value neutralization compared to BP (p = 0.0123). Furthermore, exudate dynamic over time was favorable for CAP-jet treatment.

Considering that more healings were recorded in the CAP-jet group and the informative missingness of the data for the healed wounds in wound pH-evaluation, the true effect on the pH might even be greater. CAP-jet therapy was characterized by a high local tolerability. In contrast to BP, erythema, maceration, blisters or congestion of exudate did not appear in patients treated with the CAP-jet during the study period. Furthermore, the application using the CAP-jet was well tolerated by the patients, and about half of the patients who had received the CAP-jet therapy described the procedure as comfortable.

The data presented here indicate a high potential for CAP-jet treatment as strategy for the therapy of chronic wounds independent on etiology and infection status. The CAP-jet with its fine jet facilitates high-precision handling in anatomically and pathologically demanding areas under visual control and without touch, which is unfeasible for other physical treatment methods like negative pressure, ultrasound assisted, laser based or DBD-plasma wound therapy. Benefits of the CAP-jet treatment include the effective inactivation of microorganisms, stimulation of cell proliferation and microcirculation resulting in the regeneration of destroyed tissue, acceleration of wound healing without evidence of side effects and development of resistance^25–27,29^.

Although the exact mechanism of action of CAP in the wound is currently unknown, it can be assumed that beneficial effects are based on several factors.

For instance, the reduction of the pH value in chronic wounds from alkaline to neutral milieu is prerequisite for a physiological wound healing process^30,31^. After an initial acidosis at the beginning of wound healing, pH value changes are important in the following phases to control the activity pH-dependent enzymes, such as cathepsin-G, elastase, plasmin and matrix metalloproteinases (MMP) responsible for the physiological balance of tissue degradation and reassembly processes^32,33^. MMP are involved in cell signaling, cell migration, angiogenesis and the degradation of the extracellular matrix^34^. A pH-induced imbalance between the activity of MMP and their inhibitors results in the inhibition of new tissue formation prerequisite for wound closure, and the healing process is delayed or stagnated^35–37^. Also, the high oxygen release necessary to cover the enhanced energy metabolism of regenerating cells depends on physiological pH values. Hypoxia in chronic wounds leads to a significant deceleration of healing^36,38^. Additionally, the wound pH value has an impact on bacterial colonization of wounds, as certain pathogens cannot survive within the physiological acidic milieu of intact tissue^31,33,39^.

The intact skin functions as barrier against germs^31,39^. In chronic wounds, however, the predominantly alkaline pH milieu enables pathogen growth^31,39–41^. For several bacteria it has been shown that lowering of the pH resulted in prevention or discontinuation of colony growths^31,33,42,43^. Accordingly, it has been reported that a normalization of the pH milieu in wounds supports control of wound-associated infections^15^; furthermore, a statistical significant correlation between the restoration of an acidic pH value and progress of wound healing has been observed^39,44^. Strohal et al. demonstrated in two studies the statistically highly significant correlation between the restorage of an alkaline pH and infection control as well as complete wound healing^15,45^. Therefore, the wound pH is a useful predictor for healing in chronic wounds. Independent of the ability to restore physiological pH values and thereby influence wound infection processes, CAP-jet treatment has been shown to reduce bacterial load in chronic wounds, thus further supporting healing^26,27^. In the present study, bacterial load was not analyzed; however, the effect of CAP-jet treatment on the infection score was profound, suggesting a decreasing level of pathogenic colonization in the wounds and validating the known data. While a general antimicrobial and disinfecting effect of CAP in many applications has been documented, the outcome strongly depended on the thickness of the biofilm, humidity and nutrition composition of the environment as well as the microbiome^12–14,46,47^. As a consequence, it can be assumed that the effect of CAP-jet treatment on wound infection varies depending from patients wound etiology, bacterial load, colonization, exudate level and other individual factors. In the present study, at visit 1 the number of patients with signs of infection reduced from 33.33 to 20.51% after one CAP-jet treatment and further declined at the following visits after receiving further CAP-jet treatments. This indicates, that—while a single CAP-jet application might be sufficient in some patients—subsequent treatments in other cases are required to overcome infection. However, since in the present study the bacterial load was not measured, it remains unclear whether in the patients with resolved infection signs germs were completely eradicated.

Local infection was diagnosed on the part of the investigator by using a PGA scoring system and well-established clinical criteria such as impaired fragile granulation tissue, increased exudate levels, increased pain, and impaired wound healing^48–51^. As the clinical outcome of a wound rather than the bacterial load is decisive for the healing process, this procedure was considered more relevant for the purpose of the present study^52^. Furthermore, the results of standard microbiologic detection methods do not allow any conclusion on the pathogenicity of the colonization^15,45^. The PGA is not a clinical parameter defining the development of local infection in clinical routine treatment. However, it represents an excellent study parameter as it allows the experienced investigator to assess the signs of local infection equally at the same time. As a proof of the applicability of this method, the PGA infection score stratified by visit correlated statistically significant with one of the investigated parameters, the exudate level (p = 0.0334). The assessment of the development of local wound infections with the PGA score has already been successfully applied in other studies^15,45^.

However, the assessment of exudate levels is not only relevant in terms of infection diagnosis. As a physiological part of the healing process high exudate levels are produced in the beginning of the healing process and declining in the later healing phases. Constantly high exudate levels can result in peri-wound skin damage, maceration and healing delay as well as enhanced dressing change frequency^53^. Therefore, an effective exudate management, as achieved with the CAP-jet in the present study, is prerequisite for an effective wound therapy and a sign for healing progression.

Chronic wounds are often characterized by hypoxia^54,55^. However, within the complex biochemical and physiological wound healing the oxygen demand is enhanced due to cell proliferation, immunological processes, angiogenesis and collagen synthesis^55^. With its ability to induce cell proliferation and neovascularization it can be assumed that CAP overcomes the oxygen and nutrition deficiency by improving blood supply of the wound^56^.

Taken together, data presented here confirm that CAP-jet treatment can substantially accelerate the wound healing process at different levels. The results are in line with previous CAP-jet studies^25–27^. The treatment scheme using the CAP-jet alone compared to best practice alone emphasizes the high efficacy of the CAP-jet treatment to promote wound healing even without additional best practice and/or antiseptic therapy. The jet was effective in different kinds of wounds independent of the etiology, the argon used for plasma generation ensured a constant plasma quality even with fluctuating humidity and ambient temperature.

### Study limitations and suggestions for future research

In the present RCT, the usage of the CAP-jet was compared to therapy with best practice wound care in both infected as well as non-infected chronic wounds regarding healing and tolerability. Non-inferiority and even superiority could be demonstrated with a high level of evidence for the analyzed endpoints. Within the study duration of 6 weeks, a higher percentage of completely healed wounds was achieved with CAP-jet compared to BP. Nevertheless, it has to be taken into account that it is unknown how many wounds in the control group would have healed completely within a longer observation time. However, the time frame was adequate to clearly demonstrate the favorable effect of the CAP-jet. Due to the open-label design, bias due to unblinded outcome assessment cannot be excluded, and double-blinded RCT with longer treatment durations are mandatory to confirm the presented results. Furthermore, data are derived from a relatively small population selected according to specific inclusion criteria, and baseline characteristics were not completely identical in the groups. To verify the presented results in clinical practice, studies including larger cohorts would be of use. Regarding the effect of CAP-jet treatment on local wound infection, the determination of the bacterial load additionally to the PGA score might be interesting to reveal further insights in the CAPs mode of action. In addition, further investigations with statistical analysis concerning the secondary endpoint exudate management are necessary to underpin the beneficial effects of the CAP-jet treatment versus BP.

### Conclusion

CAP-jet treatment represents a highly effective and well tolerable therapy option that is able to induce efficient and early wound healing in wounds independent of wound etiology and infection status. Based on the results that time to wound healing can be shortened under CAP-jet therapy, additional BP or antiseptic treatment was not necessary as well as that the costs for using a CAP-jet device are often lower than costs for various dressings and dressing changes required in general, hence, a medical economic aspect can be assumed.

## Methods

The present randomized controlled, open-label, non-inferiority trial (ClinicalTrials.gov Identifier: NCT04965805, 16/07/2021) was conducted at two study centers in Austria, the *Federal Academic Teaching Hospital, Feldkirch* and the *Academic Teaching Hospital, Bregenz* between April 2019 and June 2020. The study was approved by the Vorarlberg Ethics Committee according to the Austrian Medical Devices Law (Ethic Committee EK-2-13/2018-40) in compliance with the ethical guidelines of the Declaration of Helsinki (1975). Informed consent was obtained from all participants.

### Patients

Patients, who met the following inclusion criteria, were eligible for study participation: age between 18 and 95 years, presence of chronic wounds of all stages and origins defined with an onset of at least 6 weeks before study enrollment, including locally infected wounds, a wound area up to 10 × 20 cm and without visible exposure of tendon or bone.

Pregnant and breastfeeding women as well as patients with an ongoing systemic antibiotic therapy or applied within 3 weeks before start of the study were excluded from the study. Furthermore, patients with acute wounds or wounds presenting with > 30% necrotic eschar were excluded. Further exclusion criteria comprised allergy or intolerance to ingredients or excipients of CAP or to primary and secondary dressings used in the trial as well as participation in any other clinical trial up to one month prior to study start.

### Patient demographics and wound characteristics

Patient demographics and wound characteristics of each participant were evaluated and documented at baseline visit (v 0). In cases of multiple wounds, only one wound per patient was examined and assessed for further results.

### Randomization

Eligible patients were randomized in two study arms to receive either CAP-jet treatment or BP wound dressings using a permuted block design chosen with varying block sizes to maintain equally sized groups (Fig. 1). Confidentiality of the randomization sequence was ensured by keeping it in sealed and numbered envelopes.

### Interventions

Wound size, local infection, exudate level and wound pH value were assessed at baseline visit by the investigator. Furthermore, wounds were examined at day (d) 3 (v 1), d 7 ± 2 (v 2), d 10 ± 2 (v 3), d 14 ± 2 (v 4), d 21 ± 2 (v 5), d 28 ± 2 (v 6), d 35 ± 2 (v 7) and d 42 (v 8).

In the CAP-jet group, ulcers were cleaned with a 0.9% NaCl solution and covered with Gazin^®^ gauze absorbent dressing (Lohmann & Rauscher GmbH & Co. KG, Neuwied/Germany) and a secondary dressing (Peha Fix^®^, Paul Hartmann AG, Heidenheim/Germany or Mepilex XT^®^, Mölnlycke Health Care GmbH, Düsseldorf/Germany). CAP-jet treatment (kINPen^®^ Med, neoplas med GmbH, Greifswald/Germany) was performed three times in the 1st week, twice in the 2nd week and once per week in the following observation period. Treatment duration was calculated 30 s/cm^2^ wound size.

In the BP group, wounds were cleaned with a 0.9% NaCl solution or, in case of infection, with octenisept^®^ (Schülke & Mayr GmbH, Norderstedt/Germany). A wound phase-adapted primary dressing (Acticoat^®^ flex, Smith & Nephew GmbH, Hamburg/Germany; ALLEVYN™, Smith & Nephew GmbH, Hamburg/Germany; BIOSORB™, KCI Austria GmbH, Vienna, Austria; Mepilex^®^, Mölnlycke Health Care GmbH, Düsseldorf/Germany; NU-DERM^®^ and NU-GEL^®^, both KCI Austria GmbH, Vienna, Austria; Tielle and Tielle plus, both EurimPharm Arzneimittel GmbH, Saaldorf-Surheim/Germany) was used to cover non-infected wounds. Locally infected wounds were treated with antimicrobial primary dressings (octenilin^®^ Gel, Schülke & Mayr GmbH, Norderstedt/Germany; SILVERCEL^®^, Systagenix Wound Management GmbH, Hamburg/Germany). As secondary dressings, ALLEVYN™ adhesive (Smith & Nephew GmbH, Hamburg/Germany), Mepilex^®^ XT (Mölnlycke Health Care GmbH, Düsseldorf/Germany) or Peha Fix^®^ (Paul Hartmann AG, Heidenheim/Germany) were used.

In both groups, patients with VLU received additional compression therapy. Dressing changes were carried out every 2nd or 3rd (weekend) day by the clinical investigator.

### Data collection and outcome measures

#### Primary endpoint

At baseline and each subsequent visit, the total amount of granulation tissue of each wound was documented as percentage of wound area. The entire circumference of the ulcer was traced, then divided into four equal parts (one quadrant corresponding to 25%), and the amount of granulation tissue was measured using a ruler; the percentage corresponding to the amount was determined mathematically.

#### Secondary endpoints

Secondary endpoints included the reduction of wound area in cm^2^, presence of infection, wound pH value, exudate level and local tolerability.

Absolute wound area reduction was digitally assessed (CGM Clinical, CompuGroup Medical, Vienna/Austria) in an automatic manner and compared between treatment arms.

A Physician Global Assessment (PGA) score ranging from 0 (no signs of infection) to 4 (maximal signs of infection) was used to determine the infection status of the wounds by means of the well-established criteria of impaired fragile granulation tissue, more exudate, more pain, and impaired wound healing^15,49–51,57^. All wounds with scores ranging from 1 to 4 were considered as infected.

Dynamics of the pH values were measured in the wound bed with an adjusted pH-meter calibrated to pH 7 before each dressing change in the cleaned wounds at v 0, -1, -2, -4, -6, and v 8.

Exudate levels were determined at v 0, -1, -2, -3, -5, -6, and v 8 as well as quantified by the study practitioner on a scale with 0 = no exudate, 1 = weak, 2 = moderate, 3 = strong, and 4 = highly exudative.

For sensation evaluation, patients in the CAP-jet group were asked to rate their sensation of the CAP-jet administration as 1 = comfortable, 2 = no specific sensibility, 3 = uncomfortable, or 4 = strong pain before dressing change at baseline and at each visit.

Regarding safety evaluations, local tolerability was visually assessed by the investigator at each dressing change in both treatment arms based on the following parameters: presence or absence of erythema, maceration, blisters and congestion of exudate.

During the study, adverse events (AE) or serious adverse events (SAE) were evaluated and documented by the investigator according to appropriate clinical standards.

### Statistics

#### Sample size and analysis method

In the randomized controlled, open-label, non-inferiority trial it was tested whether the new wound treatment with CAP-jet was non-inferior to the current BP with a non-inferiority of maximum 15% regarding the primary endpoint sum of granulation tissue in the wound at d 42. In case of inclusion of 39 patients in each of the two treatment groups, a one-sided unpaired t-test at the 5% significance level would have a power of 83% to reject the null hypothesis that CAP-jet treatment is at least 15% worse than the BP treatment at d 42 in terms of granulation tissue formation in the wound (% of total wound area). This calculation was based on the assumption that both treatments are equally effective with respect to the primary endpoint, and that the common standard deviation is 25%. No drop-outs were expected.

#### Statistical analysis methods

All data of the per protocol (PP)-population were analyzed in the non-inferiority analyses of the primary endpoint total granulation tissue in the wound at d 42 and reduction of wound area at d 42. The PP-analyses comprised all patients who had received the randomized treatment either until complete wound healing or until end of study. Non-inferiority analyses were based on a one-sided significance level of 5%. All other analyses were conducted for the intention-to-treat (ITT)-population according to the randomized treatment based on a two-sided significance level of 5%.

The primary endpoint was tested with a non-inferiority cut-off of 15% by a one-tailed unpaired t-test with a significance level of 5%. Observed group differences were described using a two-sided 90% CI. The percentage reduction in wound area from baseline to d 42 was calculated for non-inferiority, analogous to the primary endpoint. Other secondary endpoints were tested for treatment differences. In case of normal distribution or a suitable transformation to achieve normal distribution, an unpaired t-test was performed at d 42. Otherwise, the non-parametric Wilcoxon rank sum-test was used. Time course modelling was performed for continuous data using a mixed linear model with repeated measurements (visits) per patient assuming a first order autoregressive variance–covariance matrix. In case of skewed or heteroskedastic residuals, an appropriate transformation was performed. Binary and ordinal variables were modelled over time using repeated measures (ordinal) logistic regression. Time-to-event data are graphically shown by Kaplan–Meier plot and group differences are tested by log-rank test.

Values for redundant visits after healing were imputed by 100% for granulation tissue, zero for wound area, no infection and no exudate. No values are imputed for pH. Patients’ data were also excluded for visits after the drop-out (one drop out after v 6 due to transfer to another hospital).

Statistical analyses were conducted with the SAS software version 9.4 (SAS Institute Inc., Cary, NC/USA).

## Supplementary Information

Supplementary Table S1.

## Data Availability

The datasets generated during and/or analyzed during the current study are available from the corresponding author on reasonable request.
