# Author Correction: Chronic wounds treated with cold atmospheric plasmajet versus best practice wound dressings: a multicenter, randomized, non-inferiority trial

**DOI:** 10.1038/s41598-022-11112-z

**Published:** 2022-04-25

**Authors:** R. Strohal, S. Dietrich, M. Mittlböck, G. Hämmerle

**Affiliations:** 1 Department of Dermatology, Federal Academic Teaching Hospital Feldkirch, Carinagasse 45-47, 6800 Feldkirch, Austria; 2 Central Wound Center, Department of Dermatology, Federal Academic Teaching Hospital Feldkirch, Feldkirch, Austria; 3 Center for Medical Statistics, Informatics, and Intelligent Systems, Section for Clinical Biometrics, Medical University of Vienna, Vienna, Austria; 4 Central Ambulance of Wound Care, Department of Nursing, Academic Teaching Hospital Bregenz, Bregenz, Austria

Correction to: *Scientific reports* 10.1038/s41598-022-07333-x, published online 07 March 2022

The original version of this Article contained errors in Figure 4, where the results of the bars in visit 5 and visit 6 were swapped. The original Figure 4 and accompanying legend appear below.

The original Article has been corrected.

**Figure 4:**
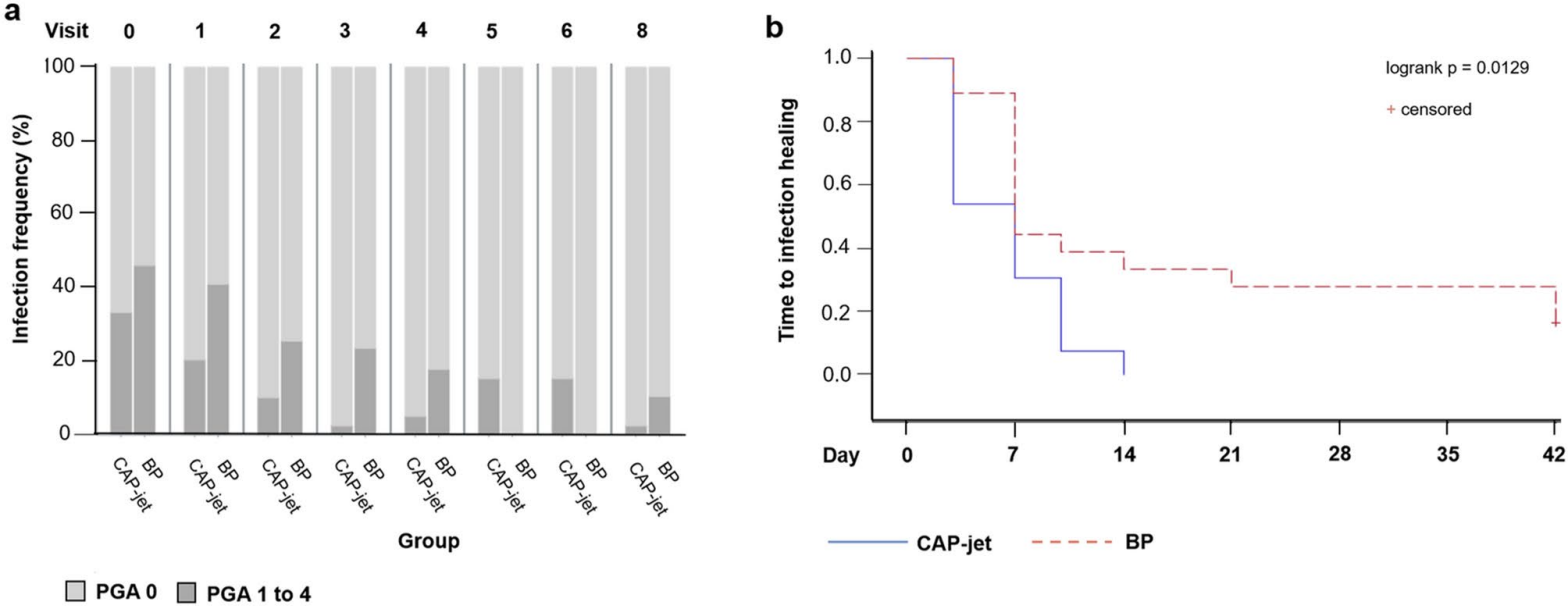
(**a**) Frequency of infection; (**b**) time to infection healing. (**a**) The newly emerged infection in CAP-jet group at v 8 was not imputed; in the BP group, one value at v 3 of one patient is missing, furthermore, the values of the patient who was transferred to another hospital after v 6 are missing in v 7 and v 8. Healed patients are considered as no infection.

