# Supplementary material for: Chronic wounds treated with cold atmospheric plasmajet versus best practice wound dressings: a multicenter, randomized, non-inferiority trial
Source: Sci Rep. 2022 Mar 7;12:3645. doi: 10.1038/s41598-022-07333-x (PMC8901692; doi:10.1038/s41598-022-07333-x)
Supplement: Supplementary file 1 — Supplementary Table S1. [file 41598_2022_7333_MOESM1_ESM.pdf]

Original Investigation

Chronic wounds treated with cold atmospheric plasmajet versus best practice wound dressings: a multicenter, randomized, non-inferiority trial

Strohal R.[1]*, Dietrich S.[2], Mittlböck M.[3], Hämmerle G.[4]

[1] Department of Dermatology, Federal Academic Teaching Hospital Feldkirch, Feldkirch, Austria

[2] Central Wound Center at the Department of Dermatology, Federal Academic Teaching Hospital Feldkirch, Feldkirch, Austria

[3] Center for Medical Statistics, Informatics, and Intelligent Systems, Section for Clinical Biometrics, Medical University of Vienna, Vienna, Austria

[4] Central Ambulance of Wound Care, Department of Nursing, Academic Teaching Hospital Bregenz, Bregenz, Austria

***Corresponding author**

Name: Prim. Univ.-Prof. Dr. Robert Strohal
Institute: Department of Dermatology,
Federal Academic Teaching Hospital Feldkirch
Address: Carinagasse 45-47,
A-6800 Feldkirch
Tel.: +43 (0)5522 303-1201
Fax: +43 (0)5522 303-7547
Email: robert.strohal@lkhf.at

Supplementary Table S1. Overview of outcomes evaluated in patients treated with CAP-jet versus BP.

	Visit (day ± allowed window)									p-value
	**0**	**1 (3)**	**2 (7 ± 2)**	**3 (10 ± 2)**	**4 (14 ± 2)**	**5 (21 ± 2)**	**6 (28 ± 2)**	**7 (35 ± 2)**	**8 (42 ± 2)**	
**Sum of granulation tissue, % (mean ± SD / median [min; max])**										
**CAP-jet**	63.08 ± 35.48<br>80.00<br>(0.00; 100.00)	77.95 ± 26.57<br>90.00<br>(20.00, 100.00)	84.1 ± 23.59<br>100.00<br>(20.00, 100.00)	87.95 ± 20.54<br>100.00<br>(30.00; 100.00)	92.05± 17.50<br>100.00<br>(20.00, 100.00)	95.13± 13.15<br>100.00<br>(30.00, 100.00)	93.33 ± 18.83<br>100.00<br>(20.00, 100.00)	95.90 ± 12.92<br>100.00<br>(40.00, 100.00)	98.97 ±3.84<br>100.00<br>(80.00; 100.00)	0.0001[#]
**BP**	45.90 ± 36.40<br>40.00<br>(0.00; 100.00)	53.59 ± 33.83<br>60.00<br>(0.00; 100.00)	57.92 ± 32.36<br>60.00<br>(0.00; 100.00)	61.54 ± 33.21<br>70.00<br>(00.00, 100.00)	63.85 ± 34.08<br>80.00<br>(10.00; 100.00)	74.62 ± 28.64<br>90.00<br>(0.00; 100.00)	74.36 ± 29.00<br>90.00<br>(10.00; 100.00)	69.87 ± 29.60<br>75.00<br>(10.00; 100.00)	77.76 ± 27.67<br>90.00<br>(20.00; 100.00)	
**Dynamics in absolute wound area, cm^2 (median [min; max])**										
**CAP-jet**	3.52<br>(0.26; 24.26)	3.24<br>(0.18; 23.94)	2.16<br>(0.16; 23.56)	1.26<br>(0.00; 18.35)	0.77<br>(0.00; 15.79)	0.42<br>(0.00; 14.62)	0.13<br>(0.00; 9.66)	0.05<br>(0.00; 5.94)	0.00<br>(0.00; 3.99)	
**BP**	3.84<br>(0.48; 45.15)	3.33<br>(0.31; 44.73)	3.16<br>(0.03; 44.36)	2.87<br>(0.03, 44.79)	2.07<br>(0.03; 45.26)	2.06<br>(0.06; 44.13)	1.76<br>(0.00; 43.91)	1.39<br>(0.00, 43.56)	1.01<br>(0.00; 38.33)	
**Relative wound area in % of baseline (mean ± SD / median [min; max])**										
**CAP-jet**	-	82.68 ±19.49<br>83.25<br>(37.50; 116.67)	66.50 ± 26.62<br>66.22<br>(7.46; 132.48)	51.31 ± 25.53<br>50.14<br>(0.00; 107.69)	38.38 ± 26.03<br>38.14<br>(0.00; 91.67)	25.77 ± 25.01<br>18.42<br>(0.00; 100.00)	15.63± 17.78<br>8.55<br>(0.00; 50.00)	10.26 ± 14.05<br>1.52<br>(0.00; 52.78)	5.32 ± 8.70<br>00.00<br>(00.00; 33.33)	< 0.0001°
**BP**	-	89.79 ± 16.79<br>95.03<br>(45.45; 124.01)	79.39 ± 26.86<br>88.73<br>(5.88; 120.79)	74.30 ±33.36<br>81.09<br>(5.88; 166.96)	65.45 ± 30.82<br>65.63<br>(5.88; 150.00)	60.64 ± 36.00<br>58.30<br>(-10.23; 192.86)	55.01 ± 39.54<br>53.91<br>(0.00; 189.29)	49.38 ± 43.03<br>44.63<br>(00.00; 200.00)	43.72 ± 43.05<br>31.66<br>(0.00; 189.29)	
**Infections, n [%]**										
**CAP-jet**	13 (33.33)	8 (20.51)	4 (10.26)	1 (2.56)	2 (5.41)	0 (0.0)	0 (0.0)	n. a.	1 (5.56)	0.0013
**BP**	18 (46.15)	16 (41.03)	10 (25.64)	9 (23.68)	7 (17.95)	6 (15.38)	6 (16.22)	n. a.	4 (11.11)	0.0002

	Visit (day ± allowed window)									p-value
	**0**	**1 (3)**	**2 (7 ± 2)**	**3 (10 ± 2)**	**4 (14 ± 2)**	**5 (21 ± 2)**	**6 (28 ± 2)**	**7 (35 ± 2)**	**8 (42 ± 2)**	
**pH (mean ± SD / median [min; max])**										
**CAP-jet**	9.95 ± 0.77<br>9.93<br>(7.36; 11.97)	9.21 ± 0.61<br>9.27<br>(7.36; 10.68)	8.92 ± 0.61<br>9.17<br>(7.35; 10.07)	n. a.	8.42 ± 0.50<br>8.33<br>(7.33; 9.51)	n. a.	8.15 ± 0.50<br>8.11<br>(7.35; 9.65)	n. a.	7.93 ± 0.48<br>8.03<br>(7.01; 8.91)	< 0.0001[#]<br>0.0123°
**BP**	10.05 ± 0.67<br>9.98<br>(9.13; 11.49)	9.81 ± 0.59<br>9.73<br>(8.21; 11.03)	9.38 ± 0.60<br>9.45<br>(8.31, 11.03)	n. a.	9.12 ± 0.62<br>9.17<br>(8.07, 10.76)	n. a.	8.94 ± 0.63<br>9.03<br>(7.65; 10.51)	n. a.	8.69 ± 0.59<br>8.60<br>(7.46; 9.95)	
**Complete healing, n (total [%])**										
**CAP-jet**	n. a.	n. a.	n. a.	2 (5.13)	1 (2.56)	8 (20.51)	5 (12.82)	3 (7.69)	4 (10.26)	
**BP**	n. a.	n. a.	n. a.	0 (0.00)	0 (0.00)	0 (0.00)	2 (5.41)	0 (0.00)	0 (0.00)	
**Local tolerability, patients with erythema, maceration, blisters and congestion of exudate, n (total [%])**										
**CAP-jet**	n. a.	0 (0.00)	0 (0.00)	0 (0.00)	0 (0.00)	0 (0.00)	0 (0.00)	0 (0.00)	0 (0.00)	
**BP**	n. a.	1 (2.56)	1 (2.56)	2 (5.13)	1 (2.56)	1 (2.56)	1 (2.56)	1 (2.78)	0 (0.00)	
**Comfortable sensation during CAP-jet treatment, n (total [%])**										
**CAP-jet**	20 (51.28)	22 (56.41)	23 (58.97)	22 (59.46)	19 (52.78)	15 (53.57)	10 (43.48)	10 (50.00)	7 (41.18)	

BP – best practice; CAP – cold atmospheric plasma; min. – minimum; max. – maximum; n – group size (number of patients with event), n. a. – not applicable; SD – standard deviation
CAP-jet group: n = 39 patients at all visits; BP group: n = 39 patients at visit 0 to 6 and n = 38 patients at visit 7 and 8 due to transfer to another hospital of one patient; healed wounds had a wound area of zero and a relative change in wound area of 100%, the number of observations for other parameters was decreased by suspended visits after diagnosed healing, additionally one missing value occurred for local tolerability at CAP-jet visit 5 and one missing value occurred for infection at BP visit 3, respectively; data for complete healing include a drop-out patient in the BP arm as non-healed patient.

[#] test for treatment differences; ° test for treatment-time interaction
